# Cohort study on 20 years’ experience of bilateral video-assisted thoracic sympathectomy (VATS) for treatment of hyperhidrosis in 2431 patients

**DOI:** 10.1590/1516-3180.2021.0078.R1.23072021

**Published:** 2022-02-21

**Authors:** Nelson Wolosker, José Ribas Milanez de Campos, Paulo Kauffman, Marcelo Fiorelli Alexandrino da Silva, Carolina Brito Faustino, Miguel Lia Tedde, Pedro Puech-Leão, Paulo Manuel Pêgo Fernandes

**Affiliations:** I MD, PhD. Full Professor, Hospital Israelita Albert Einstein (HIAE), São Paulo (SP), Brazil; and Surgeon, Department of Surgery, Vascular and Endovascular Division, Faculdade de Medicina, Universidade de Sao Paulo, Sao Paulo, SP, BR.; II MD, PhD. Surgeon, Hospital Israelita Albert Einstein (HIAE), São Paulo (SP), Brazil; and Surgeon, Department of Surgery, Thoracic Surgery Division, Faculdade de Medicina, Universidade de Sao Paulo, Sao Paulo, SP, BR.; III MD, PhD. Surgeon, Hospital Israelita Albert Einstein (HIAE), São Paulo (SP), Brazil; and Surgeon, Department of Surgery, Vascular and Endovascular Division, Faculdade de Medicina, Universidade de Sao Paulo, Sao Paulo, SP, BR.; IV MD. Surgeon, Hospital Israelita Albert Einstein (HIAE), São Paulo (SP), Brazil; and Surgeon, Department of Surgery, Vascular and Endovascular Division, Faculdade de Medicina, Universidade de Sao Paulo, Sao Paulo, SP, BR.; V MD. Surgeon, Hospital Israelita Albert Einstein (HIAE), São Paulo (SP), Brazil; and Surgeon, Department of Surgery, Vascular and Endovascular Division, Faculdade de Medicina, Universidade de Sao Paulo, Sao Paulo, SP, BR.; VI MD, PhD. Surgeon, Department of Surgery, Thoracic Surgery Division, Faculdade de Medicina, Universidade de Sao Paulo, Sao Paulo, SP, BR.; VII MD, PhD. Surgeon, Department of Surgery, Vascular and Endovascular Division, Faculdade de Medicina, Universidade de Sao Paulo, Sao Paulo, SP, BR.; VIII MD, PhD. Full Professor, Thoracic Surgery Program, Instituto do Coração (InCor), Hospital das Clínicas (HC), Faculdade de Medicina da Universidade de São Paulo (FMUSP), São Paulo (SP), Brazil; and Cardiothoracic Surgeon, Hospital Beneficência Portuguesa (BP), São Paulo (SP), Brazil.

**Keywords:** Hyperhidrosis, Quality of life, Thoracoscopy, Sympathotomy, VATS, Excessive sweating

## Abstract

**BACKGROUND:**

Primary hyperhidrosis is a condition characterized by excessive sweating, inconsistent with the needs for thermoregulation.

**OBJECTIVE:**

To assess the effectiveness and the change in the quality of life of patients undergoing bilateral VATS (video-assisted thoracoscopic sympathectomy) for treatment of hyperhidrosis, in a large case series.

**DESIGN AND SETTING:**

Cohort study conducted in a tertiary hospital specializing in hyperhidrosis located in São Paulo, Brazil.

**METHODS:**

A total of 2,431 patients who underwent surgery consisting of bilateral video-assisted thoracoscopic sympathectomy between January 2000 and February 2017 were retrospectively assessed in an outpatient clinic specializing in hyperhidrosis. The patients underwent clinical and quality of life assessments on two occasions: firstly, prior to surgery, and subsequently, one month after the operation. The presence or absence of compensatory hyperhidrosis (CH) and general satisfaction after the first postoperative month were also evaluated.

**RESULTS:**

All the patients operated had poor or very poor quality of life before surgery. In the postoperative period, an improvement in the quality of life was observed in more than 90% of the patients. Only 10.7% of the patients did not present CH, and severe CH occurred in 22.1% of the patients in this sample.

**CONCLUSION:**

Bilateral VATS is a therapeutic method that decreases the degree of sweating more than 90% of patients with palmar and axillary hyperhidrosis. It improves the quality of life for more than 90% of the patients, at the expense of development of CH in approximately 90% of the patients, but not intensely.

## INTRODUCTION

Primary hyperhidrosis (PH) is a condition characterized by excessive sweating that is inconsistent with thermoregulation needs. It has a large impact on patients’ quality of life and affects their personal and professional relationships.^1,2^ In the majority of cases, PH manifests in childhood and adolescence and persists throughout life. The typical clinical presentation is limited to the palms of the hands, the plantar region of the foot and/or the axilla, and it is symmetrical. It can also affect the head and face and often occurs in two or more regions of the body. The pathophysiology of PH is not fully understood, but it is known to result from stimulation of the sympathetic nervous system in its regulatory center. PH affects approximately 2.8% of the population, and there is a positive family history in 12.5% to 56.5% of the patients.^3^ Patients generally seek medical care later in life, and more frequently at a more financially secure age. Thus, young people end up suffering for many years before being able to receive the current well-known medical treatment.^4,5^

The initial treatment for patients with PH, until 2010, was sympathectomy. Thereafter, we began to use oxybutynin chloride as the first-line treatment. In patients for whom no adequate response to medication is attained, video-assisted thoracoscopic sympathectomy (VATS) becomes the treatment of choice.

VATS is considered to be the gold standard for the definitive treatment of hyperhidrosis. It provides excellent clinical results (reduced sweating at specific sites) and leads to a significant improvement in quality of life. These positive results are, among other causes, based on factors that are known to influence the effectiveness of sympathectomy among patients with hyperhidrosis,^1^ such as body mass index, resection level,^6,7^ preoperative quality of life^8^ and the number of resected ganglia.^9^

## OBJECTIVE

The objective of this study was to assess the effectiveness of the treatment and the change in the quality of life of patients undergoing VATS in a large case series (2,431 patients).

## METHODS

A total of 2,431 patients who underwent bilateral video-assisted thoracoscopic sympathectomy between January 2000 and February 2017 were retrospectively assessed in an outpatient clinic specializing in hyperhidrosis. This study was approved by the research ethics committee of our institution (protocol number: 1.133.255; date: June 30, 2015).

All patients treated under our care since 2000 have been routinely evaluated through regular completion of a specific medical record that includes demographic data and description of all sites of hyperhidrosis and the quantitative intensity of hyperhidrosis complaints at each site, using a questionnaire (HDSS). This is a specific quality-of-life questionnaire that was standardized by Amir et al. and translated into English by Campos et al. The quantitative degree of improvement at each point after treatment is assessed using this specific questionnaire.^10,11^

Regarding the surgical technique, in general terms, the surgery is performed with the patient in a semi-Fowler’s position at 45° to the floor. Two small incisions of approximately 1.0 cm are made in each hemithorax. The pleural cavity is accessed through an incision that is made in the fourth intercostal space, on the anterior axillary line. Through this, a 5 mm and 30° video optic is introduced. The second incision is made in the second intercostal space, on the middle axillary line, for insertion of an endoscopic scalpel. After identification of the sympathetic chain, the ganglion is isolated, always starting from the medial costal pleura, following the lateral costal pleura and ending with complete dissection of the sympathetic chain and the ganglion.

The sympathetic chain is resected on the respective costal arches. At the end of this step, the sympathetic chain segment located between the corresponding costal arches, including the target ganglion, is electrocauterized. During the sympathectomy, all patients are kept temporarily in apnea or low-flow ventilation. At the end of the surgery, the residual pneumothorax is aspirated through a nasogastric tube (no. 16) and pulmonary expansion is monitored via the video optic. The incisions are closed with intradermal sutures.

Anatomically, sympathectomy performed on the right side of the chest is slightly more laborious than on the left side owing to the greater number of large-caliber veins on the thoracic sympathetic chain and its more superior branches. This forces the surgeon to be more careful in the dissection.

Patients are usually extubated without difficulty in the operating room. After awakening from anesthesia, they are sent for anesthetic recovery and then to their room. To ensure complete pulmonary reexpansion, chest radiographs are routinely performed shortly after the surgery.

The patients underwent clinical and quality of life assessments on two occasions: firstly, prior to surgery, and subsequently, one month after the operation. All the evaluations were done by the primary investigator. The primary endpoints in the study were the following:

1. The patients’ quality of life at the first visit prior to surgery.
2. The improvement in quality of life after surgery, the clinical improvement in sweating, the presence or absence of compensatory hyperhidrosis (CH) and general satisfaction after the first postoperative month.

To measuring the degree of satisfaction, we used the quality-of-life protocol described by Amir et al., which was translated to English by Campos et al.^10,12^ Before the surgical treatment, the patients completed quality-of-life assessments without physician involvement. This protocol consists of 20 questions divided into four domains (functional-social, personal, emotional and special conditions). Five response levels are described in tables, from which only one response is allowed for each question. The patients were classified into five different levels of satisfaction, calculated as the summed total score from the protocol, ranging from 20 to 100. When the sum was greater than 84, the quality of life was considered very poor; from 69 to 83, poor; from 52 to 68, good; from 36 to 51, very good and from 20 to 35, excellent.

The improvement in quality of life after surgery was evaluated using the same protocol, and the patients were classified into five different levels of improvement, calculated as the total score from the protocol. For scores greater than 84, the quality of life was considered much worse after the surgical treatment; from 69 to 83, a little worse; from 52 to 68, equal; from 36 to 51, a little better; and from 20 to 35, much better.

The clinical improvement in sweating after treatment was defined on a quantitative scale ranging from 0 to 10, in which 0 represented no improvement and 10 represented absence of sweat, or anhidrosis, for each site of previous hyperhidrosis, based on the patients’ own assessments. From this score regarding the patients’ main complaint, the clinical improvement was graded as follows: null, 0-4; moderate, 5-7; or good, 8-10.

The degree of patient satisfaction after surgery was quantified using a questionnaire with four options for patients to describe their general satisfaction with surgery outcomes. The patients’ general satisfaction was considered to be excellent if they were 100% satisfied with the surgery outcome one month after surgery, good if they were 90% satisfied, fair if they were 75% satisfied or low if they were less than 50% satisfied.

The patients’ reports, with confirmation through physical examination, were used to analyze the incidence of CH. The severity of CH was graded as severe or non-severe. CH was considered to be severe if it was visible and embarrassing, and required more than one change of clothes during the day. CH was considered to be non-severe if it was visible and embarrassing but not enough to require a change of clothes, or if it was visible and embarrassing only sometimes (e.g. in hot weather and during exercise), or if it was present but did not bother the patient.

## RESULTS

The demographic data, main site of hyperhidrosis and the level of VATS resection in the group are presented in Table 1. The prevalence was higher among females. The mean age of the group was 24.7 years and the mean body mass index was 21.7 kg/m^2^. The main sites of hyperhidrosis were palmar and axillary. Resection of a single G3 ganglion was the most frequent procedure, followed by resection of a G4 ganglion.

**Table 1.** Demographic and technical data

	Variable	Patients	
		(n = 2431)	
**Sex (n %)**	Female	1,618	67.4%
Male	783	32.6%
**Age (years)**	Mean/SD	24.7	7.57
**BMI (kg/m^2^)**	Mean/SD	21.7	2.80
**Main site (n %)**	Palmar	1,546	64.4%
	Axillary	785	32.7%
	Plantar	29	1.2%
	Cranial-facial	42	1.7%
**Technique (n %)**	G2	100	4.2%
	G2/G3	365	15.2%
	G2/G3/G4	1	0.0%
	G3	838	35.0%
	G3/G4	264	11.0%
	G4	828	34.5%
	G4/G5	0	0.0%

BMI = body mass index; SD = standard deviation; G = ganglion.

The assessment of quality of life before surgery and the improvement in quality of life after surgery among the patients are shown in Table 2. It should be noted that all the patients operated under our care had poor or very poor quality of life before surgery. In the postoperative period, an improvement in the quality of life was observed in more than 90% of the patients.

**Table 2.** Reported pre and postoperative quality of life over the age range of the patients

		Patient group n = 2,431
Preoperative QoL [n (%)]	Excellent	0
	Very good	0
	Good	0
	Poor	630 (28.0%)
	Very poor	1617 (72.0%)
Postoperative QoL [n (%)]	Much better	1,566 (78.6%)
	A little better	321 (16.1%)
	Equal	76 (3.8%)
	A little worse	19 (0.9%)
	Much worse	10 (0.5%)

QoL = quality of life.

A review of the clinical improvement at the main site of sweating after surgery is presented in Table 3. At the palmar and axillary sites that were the main site of hyperhidrosis, more than 90% of the patients in this study reported major clinical improvement. The rate of improvement was lower (80.6%) in cases in which the cranial-facial region was the main site.

**Table 3.** Analysis on clinical improvement after surgery, at the main site of hyperhidrosis (palmar, axillary or cranial-facial)

Main site of PH	Degree of clinical improvement	Patients n = 2431
Palmar n = 1,552	High	1,465 (94.4%)
	Moderate	75 (4.8%)
	Null	12 (0.8%)
Axillary n = 765	High	692 (90.5%)
	Moderate	59 (7.7%)
	Null	14 (1.8%)
Cranial-facial n = 36	High	29 (80.6%)
	Moderate	6 (16.7%)
	Null	1 (2.8%)

PH = primary hyperhidrosis.

An analysis on the prevalence and intensity of CH is presented in Table 4. Only 10.7% of the patients did not present CH, and severe CH occurred in 22.1% of the patients in this sample.

**Table 4.** Prevalence of compensatory hyperhidrosis and its intensity

		Patients n = 2,362
Compensatory hyperhidrosis	Absent	251 (10.7%)
	Non-severe	1,588 (67.2%)
	Severe	523 (22.1%)

An analysis on the degree of satisfaction among the patients after surgery in both groups is shown in Table 5. More than 90% of the patients reported having high satisfaction with the surgery.

**Table 5.** Degree of satisfaction after the surgery

		Patients n = 2,330
Degree of postoperative satisfaction [n (%)]	100%	1586 (68.1%)
	90%	543 (23.3%)
	75%	170 (5.9%)
	< 50%	31 (1.3%)

A correlation between the technique used and presence of compensatory hyperhidrosis is shown in Table 6. Ablation at lower levels (especially G4) resulted in a lesser degree of compensatory hyperhidrosis (P < 0.00001).

**Table 6.** Correlation of technique with compensatory hyperhidrosis

Technique	Compensatory hyperhidrosis				Total	P^*^
	Absent	Mild	Moderate	Severe		
**G4**	126 (15.2%)	331 (39.9%)	249 (30%)	122 (14.7%)	**828**	
**G3 G4**	29 (10.9%)	62 (23.4%)	96 (36.3%)	77 (29.1%)	**264**	
**G3**	107 (12.7%)	271 (32.3%)	310 (36.9%)	150 (17.9%)	**838**	< 0.00001
**G2 G3**	42 (11.5%)	53 (14.5%)	140 (38.3%)	130 (35.6%)	**365**	
**G2**	7 (7%)	20 (20%)	36 (36%)	37 (37%)	**100**	

Chi-square test; G = ganglion.

## DISCUSSION

Primary hyperhidrosis is a disease that significantly affects the population, and in particular the younger population. These patients seek medical evaluations and treatments to achieve a general improvement in their quality of life.^13^

Currently in our practice, we start treatment with oxybutynin hydrochloride in all patients.^14,15,16^ This medication was proven to be effective as an initial therapy in a randomized, placebo-controlled trial by Wolosker et al., in 2012. Positive results were obtained over both the short and the long term, while the quality of life remained unchanged in situations of treatment failure.^17^ In such cases, surgical treatment was considered.^16,18^

Epidemiological evaluation of PH has revealed that there is high demand for care from young adults. It has been demonstrated that the prevalence of primary hyperhidrosis in the general population is equal between sexes.^19,20^ However, there is greater demand for treatment from females, which is due, among other reasons, to greater concern for esthetics in this group.^20,21^ This was shown in our previous study in which 67.4% of the patients were female.^22^

In our practice, we only operate on patients with a body mass index (BMI) lower than 25, since greater prevalence of severe CH has been observed among patients with a BMI greater than 25 who underwent the operation.^23^ This is why the mean BMI was 21.7 kg/m^2^ in the sample of our previous study.^22^

Regarding the distribution of the main hyperhidrosis sites, palmar hyperhidrosis was more frequent in the study group.^24^ This is usually associated with significant worsening of quality of life, given that it leads to limitations in manual activities. These data from patients who underwent operations were also observed in an epidemiological study conducted by our group in 2017.^13^ That study also showed that axillary hyperhidrosis^25^ was the second most frequent site, followed by cranial-facial and plantar sites.^26,27^ In our sample, the best results were in relation to the palmar and axillary sites. More than 90% of the patients in this study reported achieving major clinical improvement. This corroborates the indication for sympathectomy.

It has been demonstrated that the higher the level of VATS ganglion resection is, the greater the incidence of severe compensatory hyperhidrosis will be. The incidence of severe CH will also be greater if more than one ganglion level is resected in the same surgery.^6,28,29^ In addition, with higher resections, we observed higher incidence of Horner’s syndrome. Thus, we avoided extended resections in our group and gave preference to G4 or G3 ganglion sympathectomies, as demonstrated in this study, in which these were the most frequent. Earlier in our practice, resection was performed at G2 for palmar hyperhidrosis. Later on, we saved G2 in order to reduce complications. More recently, we have demonstrated that simple resection of G4 leads to therapeutic success similar to that observed with G3 resection but with a lower CH rate and maintenance of satisfaction indices with the procedure.^30^ Thus, we have shown that more inferior resections are equally effective and result in a lower possibility of side effects. The prevalence of severe compensatory hyperhidrosis was 22.1% in our sample, which was compatible with findings already reported in the literature.^31,32,33^

Success in surgical treatment of patients has been found to be independent of age,^34^ even in patients for whom there was previous clinical treatment failure.^35^ More than 95% of the patients reach moderate to high clinical improvement at the main site of hyperhidrosis.

Weng et al. retrospectively reviewed 506 patients with palmar hyperhidrosis who were treated with either R4 or R4+5, in order to evaluate the long-term results regarding postoperative moist hands (PMH) after sympathectomy.^36^ PMH occurred in over half of the patients after sympathectomy, but most of these patients were satisfied with the surgical results. Only six patients (1.3%) were dissatisfied because of frequent PMH. Those findings are consistent with our data, which showed improved quality of life in most patients. Thus, we also believe the main objective of treatment should be to improve the patient’s quality of life.

Several tools are available for assessing quality of life among hyperhidrosis patients. Wade et al. studied a wide diversity of tools.^37^ Twenty-two quality-of-life tools were identified. The most commonly used tools were the Hyperhidrosis Disease Severity Scale, the Dermatology Quality of Life Index and the Hyperhidrosis Quality-of-Life Questionnaire.

All the patients operated under our care presented poor or very poor quality of life and were preoperatively counselled regarding the risk of surgical failure and the incidence of severe compensatory hyperhidrosis after VATS. During the postoperative period, we observed improvement in the quality of life in more than 90% of the patients, and the degree of satisfaction was considered excellent or good in more than 90% of this sample. In addition, there was significant clinical improvement (greater than 90%) when the main site of hyperhidrosis was the hands or axilla. These findings reflect the patients’ expectations before and after the procedure, and demonstrate that VATS is a therapeutic method that can have a marked positive impact on the lives of patients with PH who seek treatment.

## CONCLUSION

Bilateral VATS is a therapeutic method that decreases the degree of sweating in more than 90% of the patients with palmar and axillary hyperhidrosis. It improves the quality of life of more than 90% of the patients, at the expense of development of CH in approximately 90% of patients, but not intensely.
